# Effects of oral adenosine 5'-triphosphate and adenosine in enteric-coated capsules on indomethacin-induced permeability changes in the human small intestine: a randomized cross-over study

**DOI:** 10.1186/1471-230X-7-23

**Published:** 2007-06-19

**Authors:** Martijn JL Bours, Hilde J Bos, Jon B Meddings, Robert-Jan M Brummer, Piet A van den Brandt, Pieter C Dagnelie

**Affiliations:** 1 Maastricht University, Department of Epidemiology, Nutrition and Toxicology Research Institute Maastricht, P.O. Box 616, 6200 MD Maastricht, The Netherlands; 2 University of Alberta, Department of Medicine, 2F1.12 Walter Mackenzie Health Sciences Centre, 8440 – 112 Street, T6G 2B7, Edmonton, Alberta, Canada; 3 University Hospital Maastricht, Department of Clinical Dietetics/Gastroenterology, Internal Medicine, Nutrition and Toxicology Research Institute Maastricht, P.O. Box 5800, 6202 AZ Maastricht, The Netherlands

## Abstract

**Background:**

It is well-known that nonsteroidal anti-inflammatory drugs (NSAIDs) can cause damage to the small bowel associated with disruption of mucosal barrier function. In healthy human volunteers, we showed previously that topical administration of adenosine 5'-triphosphate (ATP) by naso-intestinal tube attenuated a rise in small intestinal permeability induced by short-term challenge with the NSAID indomethacin. This finding suggested that ATP may be involved in the preservation of intestinal barrier function. Our current objective was to corroborate the favourable effect of ATP on indomethacin-induced permeability changes in healthy human volunteers when ATP is administered via enteric-coated capsules, which is a more practically feasible mode of administration. Since ATP effects may have been partly mediated through its breakdown to adenosine, effects of encapsulated adenosine were tested also.

**Methods:**

By ingesting a test drink containing 5 g lactulose and 0.5 g L-rhamnose followed by five-hour collection of total urine, small intestinal permeability was assessed in 33 healthy human volunteers by measuring the urinary lactulose/rhamnose excretion ratio. Urinary excretion of lactulose and L-rhamnose was determined by fluorescent detection high-pressure liquid chromatography (HPLC). Basal permeability of the small intestine was assessed as a control condition (no indomethacin, no ATP/adenosine). As a model of increased small intestinal permeability, two dosages of indomethacin were ingested at 10 h (75 mg) and 1 h (50 mg) before ingesting the lactulose/rhamnose test drink. At 1.5 h before indomethacin ingestion, two dosages of placebo, ATP (2 g per dosage) or adenosine (1 g per dosage) were administered via enteric-coated hydroxypropyl methylcellulose (HPMC) capsules with Eudragit^© ^L30D-55.

**Results:**

Median urinary lactulose/rhamnose excretion ratio (g/g) in the control condition was 0.032 (interquartile range: 0.022–0.044). Compared to the control condition, lactulose/rhamnose ratio after ingestion of indomethacin plus placebo was significantly increased to 0.039 (0.035–0.068); P < 0.01). The indomethacin-induced increase was neither affected by administration of encapsulated ATP (0.047 (0.033–0.065)) nor adenosine (0.050 (0.030–0.067)). Differences in L/R ratios between the conditions with indomethacin plus placebo, ATP or adenosine were not significant.

**Conclusion:**

In this study, either ATP or adenosine administered via enteric-coated capsules had no effect on indomethacin-induced small intestinal permeability changes in healthy human volunteers. The observed lack of effect of encapsulated ATP/adenosine may have been caused by opening of the enteric-coated supplement at a site distal from the indomethacin-inflicted site. Further studies on site-specific effectiveness of ATP/adenosine on intestinal permeability changes are warranted.

## Background

The intestinal mucosa on the luminal side of the gut is continuously exposed to an immense load of antigens, for instance from ingested food or resident bacteria. As a crucial part of intestinal defence mechanisms, the mucosa is involved in protecting the host against pathogenic substances. This protective function is called the intestinal barrier function [1,2]. The mucosal enterocytes are of considerable importance to this barrier function by controlling translocation of pathogenic substances.

In general, it has been proposed that there are two distinct pathways in the intestine through which translocation occurs, that is, a transcellular and a paracellular (*i.e*. intercellular) pathway [3]. The functional integrity of the paracellular pathway can be assessed by measuring gastrointestinal permeability with small saccharide markers. The use of a monosaccharide-disaccharide mixture (such as rhamnose and lactulose) is particularly useful since this provides information regarding villus tip 'damage' as a function of villus surface area [4,5].

It has been shown that increased mucosal permeability of the small intestine is associated with several gastrointestinal disorders, including inflammatory bowel disease and celiac disease [5,6]. In Crohn's disease, small intestinal permeability is thought to be positively associated with disease activity [7,8] and to be an early predictor of relapse [9-12]. In addition to disease-related changes in intestinal barrier function, several factors have been shown to negatively affect intestinal permeability, including smoking [13], alcohol intake [14,15] and use of nonsteroidal anti-inflammatory drugs (NSAIDs) [16-18].

Frequent use of NSAIDs is associated with an elevated risk of damage to the mucosal epithelium that lines the gastrointestinal tract lumen, thereby compromising integrity of the mucosal barrier. One of the earliest events in NSAID toxicity is uncoupling of oxidative phosphorylation within enterocytes resulting in depletion of cellular energy stores in the form of adenosine 5'-triphosphate (ATP), which leads to an increase in mucosal permeability in the intestine [19]. It has been demonstrated in previous experiments by Bjarnason and co-workers that mucosal permeability of the small intestine is increased within 8–10 hours after ingestion of two subsequent doses of the NSAID indomethacin (75 and 50 mg); the permeability increase is rapidly reverted, being no longer evident 48 hours after indomethacin ingestion [20-22]. Utilizing this human model of increased intestinal permeability induced by short-term challenge with indomethacin, we recently showed that topical administration of ATP into the upper small intestine attenuated the indomethacin-induced increase in intestinal permeability in healthy human volunteers [23]. In this randomized cross-over study, fasting subjects received two subsequent indomethacin dosages (75 and 50 mg) concomitant with administration of ATP or placebo directly into the upper small intestine via a naso-intestinal tube. Intestinal permeability was measured by the lactulose/rhamnose (L/R) sugar absorption test, which is a widely used and sensitive permeability measure of the small intestine [24]. Results showed that indomethacin induced an approximately two-fold increase in median urinary L/R excretion ratio relative to the basal L/R ratio in the control condition (*i.e*. no indomethacin, no ATP). Administration of ATP concomitant with indomethacin ingestion completely prevented the indomethacin-induced increase in L/R ratio [23]. This finding suggested that ATP might be a beneficial compound in alleviating detrimental NSAID effects in the small intestine.

The aim of the present study was to confirm the favourable effect of ATP on the indomethacin-induced increase in intestinal permeability, when ATP is administered via enteric-coated capsules. In addition, since the effect of ATP may have been partly mediated through its breakdown to adenosine, and since adenosine has well-known anti-inflammatory and tissue-protective effects in the intestine [25], we evaluated the effect of adenosine administered via enteric-coated capsules in the same human model of indomethacin-induced permeability changes in the small intestine.

## Methods

### Subjects

Non-smoking males and females between 18 and 30 years of age were recruited for participation at Maastricht University, The Netherlands, by way of information pamphlets. Criteria for exclusion from participation, as assessed by a short questionnaire, were: (1) history of gastrointestinal disease or current gastrointestinal disorder (*e.g*. Crohn's disease, celiac disease), (2) current use of NSAIDs (*e.g*. aspirin, ibuprofen), and (3) current use of medication which could interfere with effects of ATP/adenosine, including nucleoside transport inhibitors (*e.g*. dipyridamole, lidoflazine), non-selective adenosine receptor antagonists (*e.g*. theophylline, aminophylline), xanthine oxidase inhibitors (*e.g*. allopurinol) and antidepressant drugs. All participants received oral and written information about the aim and protocol of the study, and gave their written informed consent before participation. The study protocol was approved by the Ethics Committee of Maastricht University, The Netherlands, and carried out in compliance with the Helsinki Declaration.

### Sample size

Sample size calculation for the present randomized cross-over study was based on the results of our previous study [23], in which subjects showed an average attenuation in the indomethacin-induced increase in L/R ratio of 0.016 (*i.e*. a reduction of 33%) in response to topical ATP. In the cross-over experiments of our previous study, a standard deviation of 0.021 and correlation between paired measures of 0.74 were observed.

To be able to detect at least half of the previously observed effect, that is ~15% reduction in L/R ratio, based on the standard deviation of 0.021 and a correlation between paired measures of 0.60 (*i.e*. a conservative estimate relative to the correlation of 0.74 observed in our previous study), it was calculated that 31 subjects would be sufficient to detect a significant effect of ATP/adenosine on an indomethacin-induced increase in L/R ratio with a power of 90% and two-tailed alpha of 0.05. Accounting for potential dropout during experiments, a total of 35 participants were recruited for the present study.

### Protocol

The protocol of the present study was based on our previous study in which ATP was administered topically into the upper small intestine via a naso-intestinal tube [23]. As a model of early-stage small intestinal enteropathy, a number of experiments was performed; in each experiment, two subsequent dosages of the NSAID indomethacin (75 and 50 mg) were administered to fasting healthy human subjects. In a double-blind cross-over designed study, each subject participated in four experiments, in a randomized order and with wash-out periods of one week in between: (1) control (no indomethacin, no ATP/adenosine) as a measure of basal permeability of the small intestine, (2) indomethacin + placebo, (3) indomethacin + ATP, and (4) indomethacin + adenosine.

Since alcohol intake is known to increase intestinal permeability to larger molecules (*e.g*. lactulose) [15], and since caffeine is a non-selective adenosine receptor antagonist [26], participants were requested to abstain from alcohol and caffeine-containing beverages or foods for four days preceding and during each experiment. Also, participants were asked not to perform any kind of prolonged strenuous physical exercise (*e.g*. long-distance run, cycle race) during two days preceding each experiment, since this has been shown to increase permeability of the small intestine [27]. Antidepressants have been shown to affect activity of the enzyme adenosine deaminase [28,29], which catalyzes the breakdown of adenosine to inosine. Current antidepressant use may therefore interfere with effects of ATP/adenosine.

The control experiment comprised only the assessment of basal permeability without any intervention: after an overnight fast and after voiding, participants ingested a test drink containing 5 g lactulose (Centrafarm Services BV, Etten-Leur, The Netherlands) and 0.5 g L-rhamnose (MP Biomedicals, Aurora, OH, USA) dissolved in 100 mL water. Subsequently, total urine was collected for five hours. During the last two hours of urine collection, subjects were allowed unlimited intake of water, which stimulates adequate urine production without influencing urinary recovery of lactulose and L-rhamnose as well as the lactulose/rhamnose excretion ratio [30]. Total urine volume over the five-hour period was determined, and urine aliquots were taken and stored at -80°C until analysis.

For the experiments with indomethacin plus placebo/ATP/adenosine, at 11.5 h prior to the permeability assessment (*i.e*. ingestion of the lactulose/rhamnose test drink), participants ingested five capsules containing placebo, 2 g ATP or 1 g adenosine. One and a half hour later, participants ingested 75 mg indomethacin (Genfarma, Zaandam, The Netherlands). After an overnight fast, two and a half hours before permeability assessment, participants again ingested five capsules containing placebo, 2 g ATP or 1 g adenosine, followed by a second dose of indomethacin (50 mg) at 1 h prior to the permeability assessment. One hour later, after voiding, participants ingested the lactulose/rhamnose test drink followed by collection of total urine for five hours.

### Experimental supplements

ATP, adenosine and placebo were administered via enteric-coated hydroxypropyl methylcellulose (HPMC) Vcaps capsules, which are two-piece capsules consisting of a body and cap (a kind gift from the Laboratory of Pharmaceutical Technology, Ghent University, Ghent, Belgium). HPMC caps and bodies (capsule size 00: average weight 118 mg, volume capacity 0.91 mL and closed length 23.3 mm) were coated with Eudragit^® ^L30D-55 [31]. The enteric coating of the HPMC capsules generally dissolves within approximately 60 minutes after gastric stage at pH 6.0 and within approximately 40 minutes at pH 6.5, that is, in the proximal small intestine [31]. Using an automatic capsule-filling machine (Blokland Medical Supplies BV, IJsselstein, The Netherlands), the coated bodies were filled with lactose (BUFA BV, Uitgeest, The Netherlands), which was used as placebo as well as inert excipient, and/or ATP or adenosine and closed with the coated caps. Each participant ingested ATP by taking five capsules twice, containing a dosage of 0.4 g ATP per capsule, that is, 4 g ATP in total. Preliminary experiments demonstrated the safety of this ATP dosage (H.J. Bos, P.C. Dagnelie, unpublished observations). Adenosine was administered at an equimolar dosage: each participant ingested adenosine by taking five capsules twice, containing 0.2 g adenosine per capsule, that is, 2 g adenosine in total. The dosage of lactose that was administered does not produce symptoms of lactose intolerance, which are reported to occur at dosages of about 12 to 18 g [32].

### Intestinal permeability

Intestinal permeability was assessed using the lactulose/rhamnose (L/R) sugar absorption test: ingestion of 5 g lactulose and 0.5 g L-rhamnose dissolved in 100 mL water followed by five-hour collection of total urine. This test is based on the comparison of intestinal permeation of molecules of different sizes. The urinary L/R excretion ratio is considered to be an accurate parameter of small intestinal permeability [24]. Lactulose and L-rhamnose in collected urine samples were determined by fluorescent detection high-pressure liquid chromatography (HPLC). The method for assaying lactulose and L-rhamnose has been described previously [33]. In short, cellobiose was added to urine samples as an internal standard, and the urine was filtered through a 0.4-μm filter and diluted as necessary. Samples were deionized and then injected onto a Dionex MA-1 ion exchange column. Sugars were eluted with NaOH at a flow rate of 0.4 mL/min with concentrations ranging from 400 to 600 mmol/L. Peaks were detected using pulsed amperometric detection on a Dionex HPLC and quantified as peak areas. Calibration was performed on a daily basis with authentic standards at multiple concentrations, and the experimental standards were diluted so that the areas of all peaks fell within the calibration range.

### Statistics

Five-hour urinary excretion levels of lactulose and L-rhamnose are presented as recovery (%) of ingested lactulose and L-rhamnose, and as L/R ratios (g/g). Differences in urinary L/R ratios between different conditions were assessed using Wilcoxon signed ranks test. P-values below 0.05 were regarded statistically significant. Data are presented as Box-Whisker plots.

## Results

For the present study, a total of 35 participants were recruited, of whom two subjects did not complete all four experiments. One subject had to stop after completion of one experiment because of newly diagnosed celiac disease, and one subject only participated in two experiments due to limitation of time. Thirty-three participants (7 males, 26 females; age (mean ± SD) 22 ± 3.3 years; range 18–30 years) completed all four experiments and were included in the analyses. No side effects were reported during the experiments.

**Figure 1:**
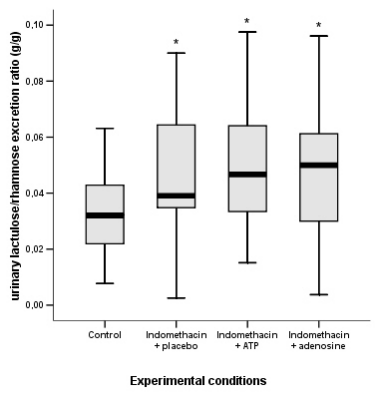
Box Whisker plot of urinary lactulose/rhamnose (L/R) excretion ratios (g/g) observed in four experimental conditions. The control condition represents basal permeability of the small intestine, as indicated by the urinary L/R ratio after ingestion of a test drink containing 5 g lactulose and 0.5 g L-rhamnose with no prior indomethacin ingestion and no placebo/ATP/adenosine challenge. The other conditions represent urinary L/R ratios after ingestion of two subsequent indomethacin dosages at 10 h (75 mg) and 1 h (50 mg) before ingestion of the test drink; at 1.5 h prior to the indomethacin dosages, two dosages of placebo (indomethacin + placebo), ATP (indomethacin + ATP, 2 g ATP per dosage) or adenosine (indomethacin + adenosine, 1 g adenosine per dosage) were administered via enteric-coated capsules (*P < 0.01 vs. control; n = 33, Wilcoxon signed ranks test). Differences in L/R ratios between the conditions with indomethacin plus placebo, ATP or adenosine were not significant.

Figure 1 shows L/R ratios of the four experimental conditions. Median L/R ratio (g/g) in the control condition (no indomethacin, no ATP/adenosine) was 0.032 (interquartile range: 0.022–0.044). After ingestion of indomethacin plus placebo, the median L/R ratio was significantly increased to 0.039 (0.035–0.068; P < 0.01 vs. control). Intake of enteric-coated capsules with either ATP or adenosine at 1.5 h prior to indomethacin ingestion had no effect on the indomethacin-induced increase in L/R ratio. Median L/R ratio after ingestion of indomethacin plus ATP was 0.047 (0.033–0.065; P = 0.22 vs. placebo), and median L/R ratio after ingestion of indomethacin plus adenosine was 0.050 (0.030–0.067; P = 0.49 vs. placebo). Median L/R ratios after indomethacin ingestion with administration of ATP or adenosine remained significantly increased compared to the L/R ratio in the control condition (P < 0.01, Fig. 1).

Table 1 shows total urine volumes (mL) and five-hour urinary recovery (%) of orally ingested lactulose (5 g) and L-rhamnose (0.5 g) in four experimental conditions. Five-hour total urine volumes were similar in all experimental conditions (Table 1). Compared to lactulose recovery in the control condition (0.14% (0.08–0.22%)), urinary lactulose recovery was significantly increased by ingestion of indomethacin plus placebo (0.20% (0.11–0.31%); P < 0.01). Neither administration of ATP nor of adenosine affected the indomethacin-induced increase in lactulose permeation. Lactulose recovery after ingestion of capsules with ATP was 0.18% (0.12–0.32%) and was 0.23% (0.11–0.28%) after ingestion of capsules with adenosine; both these values remained significantly increased compared to the control condition (P < 0.01). Basal urinary recovery of L-rhamnose was not significantly affected by ingestion of indomethacin plus placebo, ATP or adenosine (Table 1).

**Table 1:** Five-hour total urine volumes (mL) and urinary recovery (%) of orally ingested lactulose (5 g) and L-rhamnose (0.5 g) in four experimental conditions (n = 33).

**Experimental condition**	**Recovery (%) ** Median (interquartile range)		**Urine volume (mL) ** Median (interquartile range)
	
	**Lactulose**	**Rhamnose**	
**Control^1^**	0.14 (0.08–0.22)	4.41 (3.31–5.88)	298 (182–613)
**Indomethacin + placebo**	0.20 (0.11–0.31)*	4.60 (3.15–5.91)	324 (193–493)
**Indomethacin + ATP**	0.18 (0.12–0.32)*	4.12 (3.23–4.89)	371 (165–518)
**Indomethacin + adenosine**	0.23 (0.11–0.28)*	4.52 (3.90–5.26)	307 (213–563)

^1^No indomethacin, no placebo/ATP/adenosine

Differences in lactulose/rhamnose recovery and urine volumes between the conditions with indomethacin plus placebo, ATP or adenosine were not significant

*P < 0.01 vs. control (Wilcoxon signed ranks test)

In all experimental conditions, no differences were found between male and female subjects regarding urinary L/R excretion ratios, total urine volumes and urinary recovery of lactulose and L-rhamnose (data not shown).

## Discussion

We previously reported that topical administration of ATP into the human small intestine by naso-intestinal tube attenuated an indomethacin-induced increase in mucosal permeability in healthy human volunteers [23]. This finding indicated that ATP administration may be of clinical use by alleviating early adverse effects of NSAIDs and by preserving small intestinal barrier function. Like in the present study, early-phase small intestinal permeability changes were induced by administering two subsequent dosages of the NSAID indomethacin (75 and 50 mg). Short-term indomethacin challenge causes a rapid (within 8–10 h) and temporary (< 48 h) increase in intestinal permeability, which can be assessed noninvasively by calculating the urinary excretion ratio of orally ingested lactulose and L-rhamnose as markers of small intestinal permeability [20-22,34]. 

To administer ATP experimentally in the upper small intestine, the ATP in our previous study was administered via a naso-intestinal tube. Since a naso-intestinal tube is a useful way of administration in an experimental setting but not in daily practice as it can cause great discomfort to sensitive subjects or patients, the aim of the present study was to corroborate the previously observed favourable effect of ATP on indomethacin-induced permeability changes when ATP was administered via enteric-coated capsules, which is a more practically feasible mode of administration. We also evaluated the effect of adenosine administration via enteric-coated capsules, since we hypothesized that the previously observed favourable effect of ATP may have been partly mediated by its breakdown product adenosine, which has well-known anti-inflammatory and tissue-protective properties in the intestine [25].

Results of the present study show that, like in our previous study, ingestion of two subsequent indomethacin dosages significantly increases urinary L/R excretion ratio compared to the L/R ratio in the control condition, which is a measure of basal small intestinal permeability. The rise in intestinal permeability was probably due to enhanced permeation of lactulose, since urinary recovery of lactulose, but not of rhamnose, was increased by indomethacin ingestion. Unexpectedly, neither administration of encapsulated ATP nor adenosine affected the indomethacin-induced rise in small intestinal permeability, suggesting that ATP and adenosine administered via enteric-coated capsules are ineffective in attenuating an indomethacin-induced increase in paracellular permeability of the small intestinal mucosa in healthy humans.

Several potential explanations of the unexpected ineffectiveness of both enteric-coated ATP and adenosine on indomethacin-induced permeability changes in the present study can be put forward. First, the possibility should be considered that the present study results would suggest a false-positive finding in our previous study, meaning that the favourable effect of topically administered ATP on small intestinal permeability changes induced by indomethacin in our previous study was random. However, the highly significant P-value (P < 0.01) observed in our previous study, which was conducted according to a double-blind, randomized cross-over design, argues against the possibility of a false-positive finding. Second, it could be argued that subject-related differences between the present and our previous study could be responsible for the different results. This explanation is also unlikely since all study participants were recruited from the same study base (*i.e*. students at Maastricht University, The Netherlands), and age and sex ratio of the participants were quite similar in both studies. Moreover, permeability of the small intestinal mucosa of healthy subjects, as assessed by urinary excretion ratio of two different-sized test molecules, is thought to be independent of age and gender [35,36]. Nevertheless, it must be noted that intestinal permeability may vary considerably under normal conditions, since basal permeability in the present study appeared to be somewhat higher than in our previous study (median L/R ratio of 0.032 vs. 0.023, respectively) despite identical study design and methods. A third possibility is that the ATP and adenosine within the capsules were chemically unstable and therefore degraded before application. However, this is unlikely since capsules filled with ATP or adenosine in crystalline form were stored dry at 4°C, at which storage conditions both substances are stable for 24 months according to the manufacturer's specifications (BUFA BV, Uitgeest, The Netherlands). To confirm stability of the ATP used in the present study, which had been stored at 4°C for several months, we measured its quality by HPLC and found no signs of any ATP degradation (data not shown). Also, the enteric properties of the Eudragit^© ^L30D-55 coating polymer have been shown to be maintained even after 6 months of storage at different conditions [31]. A fourth potential explanation of the ineffectiveness of ATP and adenosine in the present study could be relative insolubility of ATP and adenosine. In our previous study, we administered ATP as an aqueous solution (30 mg/kg ATP dissolved in 100 mL water), whereas in the present study ATP and adenosine were administered in crystalline form within the enteric-coated HPMC capsules. However, since both ATP and adenosine are substances which are freely soluble in water [37], both are likely to dissolve within minutes in the liquid environment of the gut upon opening of the capsules. Finally, a possible explanation may be a discrepancy between the intestinal site of indomethacin-inflicted mucosal damage relative to the site of ATP/adenosine delivery from the enteric-coated capsules in the small intestine. It is believed that a short-term NSAID challenge induces permeability changes mainly in the upper small intestine (duodenum and proximal jejunum) [38,39]. This would imply that the timing of the pH-dependent release of ATP/adenosine by the enteric-coated capsules might be an important factor limiting the effectiveness of these compounds. In our previous study, in which ATP was administered as an aqueous solution via a naso-intestinal tube directly into the upper small intestine, which is the intestinal site where indomethacin-inflicted damage would occur, ATP attenuated the indomethacin-induced increase in small intestinal permeability [23]. In the present study, ATP and adenosine were administered via HPMC capsules coated with Eudragit^© ^L30D-55 [31]. Huyghebaert et al. (2004) showed by *in vitro *dissolution tests that the Eudragit^© ^L30D-55 enteric-coated capsules released 80% of their contents within 60 minutes at pH 6.0, with a lag-phase of 20 minutes after simulated gastric stage. At pH 6.5, 80% of the capsule content was released within 30 minutes after simulated gastric stage without lag-time [31]. In additional experiments by our research group, in which lithium was used as a marker to evaluate the timing of contents release by identical enteric-coated capsules ingested by healthy subjects, we observed rising lithium concentrations in plasma between approximately 90 to 200 minutes following capsule ingestion, suggesting that opening of the capsules *in vivo *may be subject to considerable variation (H.J. Bos, P.C. Dagnelie, unpublished observations). This would suggest that the lack of effect of ATP and adenosine in the present study might be explained by missing the target area of indomethacin-inflicted upper-small-intestinal damage when administering ATP and adenosine via the Eudragit^© ^L30D-55 enteric-coated capsules.

## Conclusion

In conclusion, we were not able to corroborate the previously shown beneficial effects of topical ATP administration on indomethacin-induced permeability changes in the human small intestine, when using enteric-coated capsules containing either ATP or adenosine as a more practically feasible mode of administration. The most likely explanation for the present finding is that the enteric-coated supplement may have opened at an intestinal site different (probably more distal) from the site where mucosal damage by indomethacin occurs. Further studies on site-specific effectiveness of encapsulated ATP and adenosine on intestinal permeability changes are warranted.

## Competing interests

The author(s) declare that they have no competing interests.

## Authors' contributions

MJLB contributed to developing the study protocol, performed part of the experiments, collected all data, performed data analysis, and wrote the manuscript. HJB contributed to developing the study protocol and performed part of the experiments. JBM coordinated lab analyses and helped to draft the manuscript. RJMB acted as medical backup during all experiments and helped to draft the manuscript. PAvdB assisted in developing the study protocol and helped to draft the manuscript. PCD contributed to developing the study protocol, helped to draft the manuscript and supervised all study procedures.

## Pre-publication history

The pre-publication history for this paper can be accessed here:


